# Weighted-incidence syndromic combination antibiograms to guide empiric treatment of critical care infections: a retrospective cohort study

**DOI:** 10.1186/cc13901

**Published:** 2014-05-31

**Authors:** Varinder Randhawa, Syed Sarwar, Sandra Walker, Marion Elligsen, Lesley Palmay, Nick Daneman

**Affiliations:** 1 Department of Medicine, University of Toronto, Toronto, Ontario, Canada; 2 Sunnybrook Research Institute, Toronto, Ontario, Canada; 3 Department of Pharmacy, Sunnybrook Health Sciences Centre, Toronto, Ontario, Canada; 4 Faculty of Pharmacy, University of Toronto, Toronto, Ontario, Canada; 5 Division of Infectious Diseases, Sunnybrook Health Sciences Centre, 2075 Bayview Ave, Toronto, Ontario M4N 2M5, Canada

## Abstract

**Introduction:**

Empiric antimicrobial selection for critical care infections must balance the need for timely adequate coverage with the resistance pressure exerted by broadspectrum agents. We estimated the potential of weighted incidence syndromic combination antibiograms (WISCAs) to improve time to adequate coverage for critical care infections. In contrast to traditional antibiograms, WISCAs display the likelihood of coverage for a specific infectious syndrome (rather than individual pathogens), and also take into account the potential for poly-microbial infections and the use of multi-drug regimens.

**Methods:**

Cases of ventilator-associated pneumonia (VAP) and catheter-related bloodstream infection (CRBSI) were identified over three years using stringent surveillance criteria. Based on the susceptibility profile of the culprit pathogens, we calculated the WISCA percentages of infections that would have been adequately covered by common antimicrobial(s). We then computed the excess percentage coverage offered by WISCA regimens compared to the actual antimicrobials administered to patients by 12 h, 24 h, and 48 h from culture collection.

**Results:**

Among 163 patients with critical care infection, standard practice only resulted in adequate coverage of 35% of patients by 12 h, 52% by 24 h, and 75% by 48 h. No WISCA mono-therapy regimen offered greater than 85% adequate overall coverage for VAP and CRBSI. A wide range of dual therapy regimens would have conferred greater than 90% adequate coverage, with excess coverage estimated to be as high as +56%, +42% and +18% at 12 h, 24 h and 48 h, respectively. We did not detect a decrease in mortality associated with early adequate treatment, and so could not estimate potential downstream benefits.

**Conclusions:**

WISCA-derived empiric antimicrobial regimens can be calculated for patients with intensive care unit (ICU)-acquired infections, and have the potential to reduce time to adequate treatment. Prospective research must confirm whether implementation of WISCA prescribing aids facilitate timely adequate treatment and improved ICU outcomes.

## Introduction

Several studies highlight that early provision of adequate antibiotics improves survival outcomes among critically ill patients with infection [1-3]. To ensure adequate antimicrobial treatment, clinicians often prescribe broad-spectrum empiric antibiotics while awaiting microbiology culture and susceptibility results that will enable tailoring the treatment regimen to the identified pathogen(s). Although early broad-spectrum treatment helps ensure effective treatment of infections, overuse of broad-spectrum antibiotics is driving antibiotic resistance [4]. A rise in multi-drug resistant bacteria is limiting available therapeutic options for infections in the ICU, and further reducing the likelihood that empiric treatment selections will offer adequate coverage for common ICU pathogens.

To balance the needs of adequate drug therapy with antimicrobial stewardship, Hebert *et al.* proposed the use of a novel weighted-incidence syndromic combination antibiogram (WISCA) to guide empiric treatment decisions [5]. Traditional antibiograms display the percentage susceptibility of each common bacterial pathogen to each common antimicrobial agent, but this does not help clinicians select an empiric antimicrobial regimen prior to identification of the culprit pathogen. In contrast, the WISCA displays the likelihood of adequate coverage for a specific infectious syndrome, taking into account the local weighted incidence of pathogens causing that syndrome, as well as the potential for poly-microbial infections and multi-drug regimens [5]. Hebert *et al*. demonstrated the potential of the WISCA to be more effective than standard antibiograms as an antibiotic-prescribing decision aid for patients with urinary tract infections and intra-abdominal infections [5].

The objectives of this study were to demonstrate the generation of a WISCA for critical care infections (ventilator-associated pneumonia (VAP) and catheter-related bloodstream infection (CRBSI)), to estimate the potential improvement in time to adequate empiric coverage of these infections as compared to current ICU practice if WISCAs had been used, and then to estimate the potential downstream improvements in clinical outcomes for these critically ill patients.

## Materials and methods

### Study overview and setting

A retrospective cohort study was conducted of all eligible adults diagnosed with VAP or CRBSI after admission to the ICU from May 2010 to May 2013 at Sunnybrook Health Sciences Centre (SHSC), a 700-bed university-affiliated tertiary-care hospital in Toronto, Ontario, Canada. SHSC Research Ethics Board (REB) approval was obtained for the study; the REB waived the need for informed consent, given the retrospective study design and lack of potential harms to patients. Multiple infection episodes could occur for the same patient within the study interval.

### VAP and CRBSI case definitions

The clinical syndromes were identified by active infection control surveillance over the study period, according to stringent case definitions as part of a mandatory reporting program to the Ontario Ministry of Health and Long-Term Care Critical Care Secretariat [6].

VAP was defined as a respiratory infection acquired by a patient after being on intermittent or continuous mechanical ventilation through a tracheostomy or endotracheal tube for at least 48 h that required antibiotic treatment. The patient must have had evidence of a new, worsening or persistent chest x-ray consolidation or cavitation compatible with pneumonia, associated with either white blood cell count <4,000 or >12,000, or temperature <36°C or >38°C. In addition, the case definition required: a) new onset of purulent sputum, or change in sputum character, or increase in respiratory secretions, or increase in suctioning requirements; and b) worsening gas exchange (for example, increasing oxygen needs or minute ventilation, or a worsening arterial oxygen tension/fraction of inspired oxygen (PaO_2_/FiO_2_) ratio) [6]. Culture-negative episodes of VAP were excluded from analysis, because by definition we would be unable to determine adequacy of antibiotic treatment.

CRBSI was defined as one or more positive blood cultures yielding a recognized pathogen or multiple positive blood cultures yielding a potential skin organism (for example, *Bacillus spp*., coagulase-negative *Staphylococci*, *Micrococcus spp*., *Corynebacterium spp*., or *Propionibacterium spp.*) acquired by the patient after catheter insertion for ≥48 h with no other identifiable source, along with one or more of fever ≥38°C, chills or hypotension [6]. By definition, all episodes of CRBSI are culture-positive, and so adequacy of antibiotic treatment could be determined for all cases.

### Microbiologic methods

In the SHSC microbiology laboratory, respiratory specimens from tracheal aspirates, and bronchoscopic specimens were plated on blood, bacitracin chocolate and MaConkey agar, and organism identification and susceptibilty were reported if there was moderate to heavy growth on culture, or growth above normal flora, or regardless of quantity on culture if the same pathogen was also predominant in the Gram stain. Blood cultures were performed using the BACTEC 9240 blood culture system (Becton Dickinson Diagnostic Instruments Systems, Franklin Lakes, New Jersey, USA) [7] in accordance with Clinical Laboratory Standards Institute guidelines.

### Definition of adequate empiric antibiotic treatment

The time to initiation of adequate empiric antibiotic therapy for an infection was defined as the time from culture collection to first administration of an antibiotic to which isolated pathogen(s) were all susceptible. Access was obtained to all antimicrobial susceptibility testing data performed by the laboratory, including those that were suppressed from reporting to clinicians. Intermediate susceptibility to an antibiotic was considered equivalent to resistance to that antibiotic. Where test results were not available for a specific antibiotic, antibiotic susceptibility was imputed when possible based on literature and expert review. For example, all Gram-negative bacilli were deemed resistant to vancomycin even though no testing would have been performed for this agent; similarly, all *Streptococci* were considered susceptible to linezolid, even if testing was not undertaken.

### Data sources

Study data were extracted from the Critical Care Information System (CCIS) and Stewardship Program Integrated Resource Information Technology (SPIRIT) databases, and from chart review. The CCIS contains information regarding VAP and CRBSI diagnoses, as well as multi organ dysfunction scores (MODS) as a marker of severity of illness on admission; [6] the SPIRIT database contains information regarding all culture and susceptibility data and antibiotic therapies; [8] chart review was conducted for other important covariates. We abstracted data regarding patient demographics (including age, gender, and co-morbidities), risk factors for antibiotic resistance (prior infection or colonization with antibiotic resistant organisms, long-term care residence, hospitalizations and antibiotic use within the past 90 days, and duration of hospital stay prior to infection onset) and outcomes (post-infection length of hospital and ICU stay, and ICU and hospital mortality).

### Deriving a weighted-incidence syndromic combined antibiogram (WISCA)

A WISCA was calculated based on pathogens isolated from patients with VAP, CRBSI, or either of these critical care associated infections [5]. In contrast to usual antibiograms, which reflect the percent susceptibility of individual organisms to individual antibiotics, the WISCA provides a weighted susceptibility of all organisms causing a given infectious syndrome. The WISCA also provides the opportunity to account for poly-microbial cultures and multi-drug treatment regimens. We elected to study the most common Gram-positive and Gram-negative antimicrobial agents available on our hospital ICU formulary and for which automated sensitivity testing is routinely performed (ciprofloxacin, tobramycin, ceftriaxone, ceftazidime, piperacillin-tazobactam, ertapenem, meropenem, cloxacillin, vancomycin and linezolid). We also examined all dual combinations of these agents other than those which involved use of double beta-lactam agents or redundant Gram-positive coverage. For each case of VAP or CRBSI, we determined which of these monotherapy and dual-therapy regimens would have provided adequate coverage (as defined above) for the isolated pathogen(s). The final WISCA displays the overall percentage of infection episodes that would have been adequately covered by each monotherapy and dual-therapy regimen.

### Statistical analysis

We calculated the excess percentage of adequate coverage that would have been achievable with each monotherapy and dual-therapy WISCA regimen, if it had been used in place of the actual empiric antimicrobials used in this retrospective cohort (at time points of 12 h, 24 h and 48 h from culture collection). For any specific mono or dual-therapy regimen excess percentage of coverage was calculated as follows:

Estimated excess percentage of coverage at a specific time point = (% of infections that would have been covered by that regimen if it had been selected empirically) – (Observed% adequately covered by actual empiric selections used in retrospective cohort).

In the primary analysis, we calculated these excess percentages of adequate coverage for the overall cohort of patients with critical care infection (either VAP or CRBSI) to simulate the empiric situation when the site of infection is unknown. We then recalculated the excess percentages of adequate coverage separately for the VAP and CRBSI subgroups.

Patients were dichotomized according to whether they had received any early adequate empiric treatment (within 24 h of culture collection) or had not received any adequate treatment during this time interval. A 24-h cut point was selected as this almost always precedes the availability of final susceptibility testing results. The baseline characteristics and outcome measures were compared among those receiving versus those not receiving adequate treatment (chi-square test of proportions, and Wilcoxon rank sum test for continuous variables). In sensitivity analyses, the impact of early adequate treatment on hospital mortality was also compared amongst VAP and CRBSI subgroups, and by applying variations in the definition of time to adequate treatment (12-h, 24-h, 48-h, and 72-h cut points). If we detected a reduction in mortality with early adequate treatment, we planned to extrapolate the potential mortality reduction achievable with various WISCA regimens that offered greater adequate empiric coverage rates than that achieved with usual care in this retrospective cohort: relative risk reduction with adequate treatment × (% excess coverage with WISCA regimen). We also calculated the percentage of adequate coverage offered by the subset of dual-therapy regimens that would have been considered concordant with the Canadian VAP treatment guidelines [9] or the Infectious Diseases Society of America (IDSA) CRBSI management guidelines [10].

### Sample size calculation

With our expected available sample of approximately 150 patients with critical care infections, we calculated that we would be able to estimate the proportion of those receiving adequate therapy in the setting of usual care with a precision of ± 6% (with alpha 0.05, 95% confidence level, estimated true proportion of 50%). Similarly, we would be able to estimate the proportion of adequate coverage offered by each mono or dual-therapy WISCA regimen with a precision of ± 6% (and estimates would be even more precise if true proportions were >50%). The impact of adequate treatment on mortality was exploratory because we determined that we would only have adequate power to detect a 25% absolute risk reduction in this outcome (assuming 80% received adequate treatment, baseline mortality of 40%, power ≥80%, and alpha 0.05).

## Results

During the 3-year study period, 163 critical care infections were identified, including 107 culture-positive cases of VAP and 56 CRBSI episodes.

### Receipt of adequate empiric antibiotic therapy

**Figure 1:**
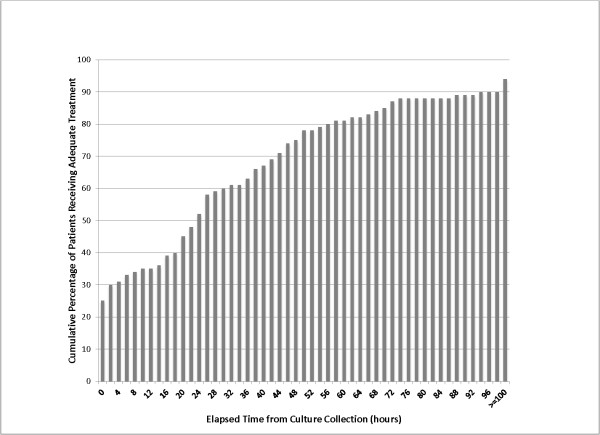
**Time to first adequate antimicrobial treatment for critical care infection.** The gray bars represent the cumulative percentage of patients with ventilator-associated pneumonia or catheter-related bloodstream infection receiving adequate empiric antimicrobial treatment as a function of time from index microbiology specimen collection.

Figure 1 highlights the time from culture collection to the receipt of adequate empiric antibiotics in critically ill patients with VAP or CRBSI. At 12 h following culture collection, approximately one third (35%) of patients had received adequate antibiotic coverage to which the isolated pathogen(s) were all susceptible; by 24 h about half (52%) had received adequate antibiotics; by 48 h three-quarters (75%) had received adequate antibiotics; and only by 96 hours had this cumulative percentage increased to 90%.

### Baseline characteristics of patients receiving or not receiving early adequate antibiotic treatment

The baseline characteristics were similar among critically ill patients receiving early adequate treatment within the first 24 h (n = 84) versus those not receiving early adequate treatment (n = 79) (Table 1). Those not receiving early adequate treatment had longer lengths of stay prior to infection (12 (7 to 47) days versus 10 (15 to 16), *P* = 0.03). Those not receiving adequate treatment were also older, but this difference was not statistically significant (64.1 (IQR 46 to 76) versus 56 (36 to 75 years), *P* = 0.09). Otherwise, there were no significant differences in patient demographics, comorbidities, severity of illness, or culprit pathogens (Table 1).

**Table 1:** Characteristics of patients with critical care infections receiving versus not receiving adequate treatment within 24 hours

**Characteristics**	**Patients receiving early adequate treatment within 24 h (n = 84)**	**Patients not receiving early adequate treatment within 24 h (n = 79)**	***P*-value**
Age, years, median (IQR)	56 (36 to 75)	64.13 (46.25 to 76)	0.09
Male sex, n (%)	63 (75%)	57 (72%)	0.72
Admission source, n (%)			
Emergency Room	31 (37%)	25 (32%)	0.51
Ward	14 (17%)	21 (27%)	0.13
Operating Room	17 (20%)	11 (14%)	0.31
Step-up	6 (7%)	8 (10%)	0.58
Other	16 (19%)	14 (18%)	0.84
ICU Type, n (%)			
Medical	28 (33%)	25 (32%)	0.87
Surgical	0 (0%)	1 (1.3%)	0.48
Trauma	2 (2%)	5 (6%)	0.27
Burns	9 (11%)	7 (8.7%)	0.79
Neurosurgical	36 (43%)	30 (38%)	0.63
Cardiac	9 (11%)	11 (14%)	0.64
ICU level, n (%)			
Level 3	84 (100%)	79 (100%)	1.00
Comorbidities, n (%)	66 (78.6%)	62 (78.5%)	1.00
Heart disease	25 (30%)	16 (20%)	0.21
Peripheral vascular	9 (10.8%)	6 (8%)	0.59
Diabetes	16 (19%)	17 (21%)	0.70
Renal disease/dialysis	13 (15.5%)	10(13%)	0.66
Hypertension	27 (32%)	29 (36%)	0.62
COPD/asthma	12 (14%)	6 (8%)	0.22
Lung disease	7 (8.3%)	11 (14%)	0.32
Gastrointestinal disease	10 (12%)	15 (19%)	0.28
Liver disease	2 (2.4%)	6 (8%)	0.16
Neurological disease	14 (16.7%)	15 (19%)	0.84
Solid organ cancer	13(15.5%)	12 (15%)	1.00
Leukemia/lymphoma	3 (11.1%)	3 (4%)	1.00
Chemotherapy/radiation	4 (15.5%)	8 (10%)	0.24
Surgery	10 (12%)	18 (23%)	0.10
Infection	2(2.4%)	7 (9%)	0.09
Multi organ dysfunction score, median (IQR)	4 (2 to 6.2)	5 (3 to 6.5)	0.76
Organisms, n (%)			
Mono-microbial	59 (70%)	58 (73.4%)	0.73
Poly-microbial	25 (30%)	21 (26.6%)	0.73
Frequent organisms, n (%)			
*Streptococcus pneumoniae*	3 (3.6%)	8 (10%)	0.12
*Staphylococcus aureus*	19 (23%)	17 (21.5%)	1.00
*Serratia marcescens*	2 (2.4%)	3 (4%)	0.67
*Pseudomonas aeruginosa*	22 (27%)	12 (15%)	0.12
*Klebsiella pneumonia*	5 (6%)	9 (11.5%)	0.27
*Klebsiella oxytoca*	5 (6%)	2 (2.5%)	0.44
*Haemophilus influenzae*	10 (12%)	5 (6.3%)	0.28
*Escherichia coli*	8 (9.5%)	1 (1.4%)	0.03
*Enterococcus faecalis*	3 (3.6%)	5 (6.3%)	0.49
*Enterobacter cloacae*	3 (3.6%)	5 (6.3%)	0.49
*Enterobacter aerogenes*	4 (4.8%)	5 (6.3%)	0.74
Coagualse-negative staphylococci	7 (8.3%)	14 (17.7%)	0.10
*Citrobacter koseri*	5 (6%)	0 (0%)	0.06
Other	13 (15.5%)	18 (22.8%)	0.32
Average duration of hospital admission prior to infection, days, median (IQR)	10 (5–16) Days	12 (7 to 46.5)	0.03
Previous antibiotic use in the past 90 days before hospital admission, n (%)	7 (8.4%)	8 (10%)	0.79
Previous hospital visit in the past 90 days before hospital admission, n (%)	13 (15.5%)	13 (16.5%)	1.00

COPD, chronic obstructive pulmonary disease.

### Outcomes associated with receipt of early adequate treatment among patients with critical care infections

Hospital survival was similar among patients receiving adequate treatment within 24 h (60/84, 71%) versus those that did not receive this early adequate treatment (55/79, 70%) (*P* = 0.86) (Table 2). Early administration of adequate antibiotic therapy was not associated with ICU survival, hospital or ICU length of stay, or length of mechanical ventilation or pressor use (Table 2). In a sensitivity analysis, we explored wider variations in the definition of time to adequate antibiotics (using time cut offs of 12 h, 24 h, 48 h and 72 h). At none of these cut-points did we detect a statistically significant association between adequate treatment and mortality (data not shown). A multivariate analysis adjusting for patient and pathogen factors did not detect an association of early adequate treatment and mortality (data not shown).

**Table 2:** Outcomes of patients with critical care infections receiving vs not receiving early adequate treatment within 24 hours

**Outcomes**	**Patients receiving early adequate treatment within 24 h (n = 84)**	**Patients not receiving early adequate treatment within 24 h (n = 79)**	***P*-Value**
Hospital survival, n (%)	60 (71%)	55 (70%)	0.86
ICU survival, n (%)	66 (79%)	63 (80%)	1.00
Hospital length of stay, days, median (IQR)	57 (31 to 92)	55 (34.25 to 128.75)	0.25
ICU length of stay, days, median (IQR)	23 (15 to 46)	28.5 (16.25 to 47.75)	0.62
Post infection hospital, days, median (IQR)	36 (18 to 69)	39 (17.5 to 80)	0.99
Length of mechanical ventilation, days, median (IQR)	18 (10 to 37)	17 (11 to 38.25)	0.78
Length of pressor use, days, median (IQR)	5 (0 to 20)	5 (0 to 27)	0.96

### Potential improvement in time to adequate empiric antibiotic coverage for critical care infections with the use of WISCAs

A WISCA was calculated to determine the percent coverage that would have been provided by common monotherapy or dual-combination anti-microbial regimens for critical care infections (Table 3). We also estimated the potential excess coverage achievable by these WISCA regimens in comparison to the antibiotic regimens actually administered to the retrospective cohort of critically ill patients.

**Table 3:** Potential improvement in adequacy of empiric coverage for patients with critical care infections at 12, 24, and 48 hours using WISCA empiric regimens

**WISCA empiric regimens**	**Percentage of VAP or CRBSI with documented adequate coverage by this regimen (n = 163)**	**Excess percentage of adequate coverage compared to retrospective cohort**		
		**12 h**	**24 h**	**48 h**
**Monotherapy**				
Ciprofloxacin	110 (67%)	+30%	+16%	-8%
Tobramycin	75 (46%)	+9%	-5%	-29%
Ceftriaxone	77 (47%)	+10%	-4%	-28%
Ceftazidime	77 (47%)	+10%	-4%	-28%
Pip.-Tazo.	103 (63%)	+26%	+12%	-12%
Ertapenem	93 (57%)	+20%	+6%	-18%
Meropenem	121 (74%)	+37%	+23%	-3%
Cloxacillin	30 (18%)	-19%	-33%	-57%
Vancomycin	67 (41%)	+5%	-10%	-34%
Linezolid	68 (42%)	+6%	-9%	-33%
**Dual combination therapy**				
Meropenem + Vancomycin	152 (93%)	+56%	+42%	+18%
Ertapenem + Vancomycin	127 (78%)	+41%	+27%	+3%
Pip.-Tazo. + Vancomycin	144 (88%)	+51%	+37%	+13%
Ceftazidime + Vancomycin	141 (87%)	+50%	+36%	+12%
Ceftriaxone + Vancomycin	117 (72%)	+35%	+21%	-3%
Ciprofloxacin + Vancomycin	151 (93%)	+56%	+42%	+18%
Tobramycin + Vancomycin	151 (93%)	+56%	+42%	+18%
Ciprofloxacin + Cloxacillin	134 (82%)	+45%	+31%	+7%
Tobramycin + Cloxacillin	116 (71%)	+34%	+20%	-4%
Meropenem + Tobramycin	126 (77%)	+40%	+26%	+2%
Ertapenem + Tobramycin	126 (77%)	+40%	+26%	+2%
Pip.-Tazo. + Tobramycin	128 (79%)	+42%	+28%	+4%
Ceftazidime + Tobramycin	102 (63%)	+26%	+12%	-12%
Ceftriaxone + Tobramycin	115 (71%)	+34%	+20%	-4%
Meropenem + Ciprofloxacin	143 (88%)	+51%	+37%	+13%
Ertapenem + Ciprofloxacin	143 (88%)	+51%	+37%	+13%
Pip.-Tazo. + Ciprofloxacin	144 (88%)	+51%	+37%	+13%
Ceftazidime + Ciprofloxacin	137 (84%)	+47%	+33%	+9%
Ceftriaxone + Ciprofloxacin	136 (83%)	+46%	+32%	+8%
Ciprofloxacin + Tobramycin	135 (82%)	+45%	+31%	+7%

WISCA, weighted incidence syndromic combined antibiogram; VAP, ventilator-associated pneumonia; CRBSI, catheter-related bloodstream infection; Pip.-Tazo., piperacillin-tazobactam.

In this study, no monotherapy WISCA regimen would have provided >80% adequate coverage for both VAP and CRBSI. Meropenem would have offered the greatest degree of adequate coverage (74%), followed by ciprofloxacin (67%) and piperacillin-tazobactam (63%). By 24 h, most monotherapy WISCA regimens provided less adequate coverage than that achieved in the retrospective cohort. However, many dual-antibiotic WISCA regimens would have provided >80% adequate coverage (particularly when a Gram-negative agent was administered together with vancomycin), and a few even afforded >90% adequate coverage. At 24 h, all combinations still offered a greater probability of adequate coverage than that obtained in the retrospective cohort. There was no excess coverage provided by the monotherapy regimens at 48 h, but there was some excess adequate coverage for many dual-antimicrobial regimens at this late time point (Table 3).

Similar WISCAs were derived separately for the VAP and CRBSI subgroups (Tables 4 and 5). In these subgroup analyses, there were still no monotherapy WISCA regimens that offered >85% adequate antibiotic coverage; in fact, for CRBSI, there were no monotherapy regimens that offered >60% adequate coverage. In contrast, a greater number of dual-therapy regimens afforded >90% adequate coverage for either the VAP or CRBSI sub-groups. Dual combination regimens involving two Gram-negative agents maximized the percentage of adequate antibiotic coverage for VAP, while those involving vancomycin plus an agent with broad Gram-negative coverage maximized excess antibiotic coverage for CRBSI.

**Table 4:** Potential improvement in adequacy of empiric coverage for ventilator-associated pneumonia at 12 h, 24 h, and 48 h using WISCA empiric regimens

**WISCA empiric regimens**	**Percentage of VAP with documented adequate coverage by this regimen (n = 107)**	**Excess percentage of adequate coverage compared to retrospective cohort**		
		**12 h**	**24 h**	**48 h**
**Monotherapy**				
Ciprofloxacin	82 (77%)	+37%	+24%	0%
Tobramycin	55 (51%)	+11%	-3%	-26%
Ceftriaxone	59 (55%)	+15%	+2%	-22%
Ceftazidime	58 (54%)	+14%	+1%	-23%
Pip.-Tazo.	74 (69%)	+29%	+16%	-8%
Ertapenem	70 (65%)	+25%	+12%	-12%
Meropenem	91 (85%)	+45%	+32%	+8%
Cloxacillin	29 (27%)	-13%	-26%	-50%
Vancomycin	36 (34%)	-6%	-19%	-43%
Linezolid	36 (34%)	-6%	-19%	-43%
**Dual combination therapy**				
Meropenem + Vancomycin	99 (93%)	+53%	+40%	+15%
Ertapenem + Vancomycin	77 (72%)	+32%	+19%	-5%
Pip.-Tazo. + Vancomycin	92 (86%)	+46%	+33%	+9%
Ceftazidime + Vancomycin	89 (83%)	+43%	+30%	+6%
Ceftriaxone + Vancomycin	68 (64%)	+24%	+11%	-13%
Ciprofloxacin + Vancomycin	101 (94%)	+54%	+41%	+17%
Tobramycin + Vancomycin	99 (93%)	+53%	+40%	+16%
Ciprofloxacin + Cloxacillin	99 (93%)	+53%	+40%	+16%
Tobramycin + Cloxacillin	93 (87%)	+47%	+34%	+10%
Meropenem + Tobramycin^*^	94 (88%)	+48%	+35%	+11%
Ertapenem + Tobramycin	93(87%)	+47%	+34%	+10%
Pip.-Tazo. + Tobramycin^*^	96 (90%)	+50%	+37%	+13%
Ceftazidime + Tobramycin^*^	79 (74%)	+14%	+21%	-3%
Ceftriaxone + Tobramycin	92 (86%)	+46%	+33%	+9%
Meropenem + Ciprofloxacin^*^	101 (94%)	+54%	+41%	+17%
Ertapenem + Ciprofloxacin	100 (93%)	+53%	+40%	+16%
Pip.-Tazo. + Ciprofloxacin^*^	102 (95%)	+55%	+42%	+18%
Ceftazidime + Ciprofloxacin^*^	100 (93%)	+53%	+40%	+16%
Ceftriaxone + Ciprofloxacin	99 (93%)	+53%	+40%	+16%
Ciprofloxacin + Tobramycin	99 (93%)	+53%	+40%	+16%

WISCA, weighted incidence syndromic combined antibiogram; VAP, ventilator-associated pneumonia; CRBSI, catheter-related bloodstream infection; Pip.-Tazo., piperacillin-tazobactam. *Regimens that would be considered concordant with Canadian VAP treatment guidelines [9].

**Table 5:** Potential improvement in adequacy of empiric coverage for catheter-related bloodstream infection at 12 h, 24 h, and 48 h using WISCA empiric regimens

**WISCA empiric regimens**	**Percentage of CRBSI with documented adequate coverage by this regimen (n = 56)**	**Excess percentage of adequate coverage compared to retrospective cohort**		
		**12 h**	**24 h**	**48 h**
**Monotherapy**				
Ciprofloxacin	28 (50%)	+20%	+2%	-21%
Tobramycin	20 (36%)	+6%	-12%	-35%
Ceftriaxone	18 (32%)	+2%	-16%	-39%
Ceftazidime	19 (34%)	+4%	-13%	-37%
Pip.-tazo.	29 (52%)	+22%	+4%	-19%
Ertapenem	23 (41%)	+11%	-7%	-30%
Meropenem	30 (54%)	+24%	+6%	-17%
Cloxacillin	1 (2%)	-28%	-47%	-69%
Vancomycin	31 (55%)	+25%	+7%	-16%
Linezolid	32 (57%)	+27%	+9%	-14%
**Dual-combination therapy**				
Meropenem + Vancomycin^*^	53 (95%)	+65%	+47%	+24%
Ertapenem + Vancomycin	50 (89%)	+59%	+42%	+18%
Pip.-Tazo. + Vancomycin^*^	52 (93%)	+63%	+45%	+22%
Ceftazidime + Vancomycin^*^	52 (93%)	+63%	+45%	+22%
Ceftriaxone + Vancomycin	49 (88%)	+58%	+40%	+17%
Ciprofloxacin + Vancomycin	52 (93%)	+63%	+45%	+22%
Tobramycin + Vancomycin	50 (89%)	+59%	+41%	+18%
Ciprofloxacin + Cloxacillin	35 (63%)	+33%	+15%	-8%
Tobramycin + Cloxacillin	23 (41%)	+11%	-7%	-30%
Meropenem + Tobramycin	32 (57%)	+27%	+9%	-14%
Ertapenem + Tobramycin	33 (59%)	+29%	+11%	-12%
Pip.-Tazo. + Tobramycin	32 (57%)	+27%	+9%	-14%
Ceftazidime + Tobramycin	23 (41%)	+11%	-7%	-30%
Ceftriaxone + Tobramycin	23 (41%)	+11%	-7%	-30%
Meropenem + Ciprofloxacin	42 (75%)	+45%	+27%	+4%
Ertapenem + Ciprofloxacin	43 (77%)	+47%	+29%	+6%
Pip.-Tazo. + Ciprofloxacin	42 (75%)	+45%	+27%	+4%
Ceftazidime + Ciprofloxacin	37 (66%)	+36%	+18%	-5%
Ceftriaxone + Ciprofloxacin	37 (66%)	+36%	+18%	-5%
Ciprofloxacin + Tobramycin	36 (64%)	+34%	+16%	-7%

WISCA, weighted incidence syndromic combined antibiogram; VAP, ventilator-associated pneumonia; CRBSI, catheter-related bloodstream infection; Pip.-Tazo., piperacillin-tazobactam. *Regimens that would be considered concordant with Infectious Diseases Society of America CRBSI treatment guidelines.

Among the VAP WISCA dual-therapy regimens we studied, 6 of 20 would be considered concordant with the Canadian VAP treatment guidelines (Table 4 footnote). These 6 regimens offered an average rate of adequate coverage of 89%, but this ranged from a minimum of 74% for ceftazidime/tobramycin to a maximum of 95% average coverage for piperacillin-tazobactam/ciprofloxacin. Among the 14 non-guideline concordant dual-therapy regimens studied, the average rate of adequate coverage for VAP was 87%, and many offered rates of adequate coverage similar to the guideline concordant regimens (Table 4). Among, the CRBSI WISCA dual-therapy regimens we studied, 3 of 20 would be considered concordant with IDSA CRBSI treatment guidelines (Table 5 footnote). These three regimens offered the highest rates of adequate coverage (93% to 95%), and only one non-guideline concordant regimen (vancomycin/ciprofloxacin) offered as high a likelihood of adequate coverage (93%) (Table 4).

## Discussion

In this study we have demonstrated the generation of a WISCA as a tool to predict the likelihood of adequate empiric antimicrobial coverage for patients with critical care infections. The WISCA helped illuminate the poor likelihood of coverage by all empiric monotherapy regimens for VAP or CRBSI, while revealing a variety of dual-therapy regimens offering high probabilities of adequate coverage for either or both of these infectious foci. Many of the dual-combination therapies that would have been recommended by WISCA-guided data offer a much higher degree of adequate antibiotic coverage than that achieved by 12 h, 24 h, and even 48 h in our retrospective patient cohort, and so could potentially decrease time to adequate antimicrobial treatment in this vulnerable patient population. However, we detected no association between timing of adequate treatment and patient outcomes in our ICU, and so we were unable to estimate the potential impact of WISCA use on length of stay and mortality among patients with these critical care infections.

The low rates of adequate empiric coverage in our cohort (only 1/2 of patients by 24 h and only 3/4 of patients by 48 h) are in line with the findings of previous investigations of VAP treatment [2,3] These low rates of coverage are a function of the high rates of antimicrobial resistant pathogens in North America, particularly within the confines of the ICU [11,12]. ICU antimicrobial resistance surveillance studies in Canada [12] and the US [11] report high percentages of antimicrobial resistance, but like traditional hospital antibiograms they display these proportions for individual bug-drug combinations (for example, 30% of Pseudomonas isolates in US ICUs were found to be resistant to ciprofloxacin in 2009 to 2010) [11]. These national, regional or hospital-specific antibiogram reports can alert clinicians to the general problem of antimicrobial resistance, but do not answer the immediate clinical question for their patients; namely, what is the likelihood of adequate coverage that will be achieved with each empiric treatment option for a patient presenting with clinical infection. By displaying the susceptibility rates by syndrome, rather than pathogen, the WISCA has the potential to increase adequate antimicrobial use. In our population, there were WISCA-guided regimens that would have offered an excess of up to +42% more patients receiving adequate coverage by 24 h compared to current practice.

According to WISCA calculations, the regimens with the highest likelihood of adequate coverage for CRBSI were the same as those that would be considered concordant with IDSA CRBSI treatment guidelines by containing both vancomycin and an anti-Pseudomonal beta-lactam [10]. Therefore, it is not clear whether locally derived WISCA data would improve upon the likelihood of adequate CRBSI coverage, if practice was already concordant with these guidelines. In contrast, WISCA calculations for VAP indicated that many non-guideline concordant dual-therapy regimens were just as likely as guideline concordant dual-therapy regimens to provide adequate VAP coverage [9]. Therefore, WISCA data has the potential to expand the range of empiric treatment options for VAP patients based on local antimicrobial resistance profiles.

Our study was unable to confirm the clinical benefit of early adequate antibiotic treatment, that has been documented in previous studies of infection in critically ill patients [1-3,13-15]. Although the importance of early adequate treatment is controversial [16-18], we believe that the lack of association with mortality in our study related to insufficient statistical power. The clinical trajectory of only a subset of patients with critical care infection would be expected to benefit from early adequate treatment. Many patients with infection-triggered systemic inflammation will continue to deteriorate even once the trigger has been treated; whereas other non-hypotensive patients may be stable enough to tolerate substantial delays in treatment [19], A recent meta-analysis has determined that the attributable mortality of VAP is only 13%, which represents a low ceiling on the potential benefit of any therapeutic intervention including timely antibiotics [20]. Based on the observed 30% mortality in our population, we would have required 332 patients to detect such a maximal 13% absolute mortality reduction; to detect a more realistic mortality reduction of 5% a prospective study would need to enroll approximately 2,500 patients.

Even though infections were prospectively defined using stringent surveillance definitions, over-diagnosis of VAP likely remained important given the lack of an effective gold standard test for this condition, the notoriously low specificity of clinical and microbiology criteria, and the pervasiveness of competing diagnoses such as acute respiratory distress syndrome and congestive heart failure [21-25]. Inclusion of patients without VAP may have diluted any signal of benefit of early antibiotic treatment. Bias-by-indication may also have masked a benefit of adequate treatment, given that broader treatment approaches may have been more commonly provided to sicker patients; although we accounted for important measurable confounders, we cannot rule out residual confounding in this observational study design. Our study was also limited by the use of single-center data, such that the types of patients and prevalence of antimicrobial resistance may differ from those in other centers, but we have demonstrated how WISCAs could easily be generated by other ICUs based on their local ecology. By definition, WISCA values and adequate treatment definitions are derived from culture-positive patients, so we cannot necessarily extrapolate our findings to culture-negative infections in the ICU. Additionally, the predominant use of relatively non-specific endotracheal tube specimens means that the culprit organism(s), and in turn the adequacy of treatment, may have been mislabeled in some VAP cases. We defined time to adequate antibiotics from the time of culture collection, whereas time from onset of hypotension or organ failure may be more closely tied to important patient outcomes [1]. Lastly, the WISCA does not quantify the hazards of broad-spectrum antimicrobial use, which must also be weighed into empiric antibiotic decisions.

## Conclusions

In summary, our study demonstrated that a WISCA can be derived for patients with ICU-acquired infections, and that WISCA-derived empiric antimicrobial regimens have the potential to reduce the time to initiation of adequate treatment. Prospective research is needed to confirm whether actual provision of WISCA antimicrobial prescribing-aids to ICU clinicians are associated with improvements in timely and rational antimicrobial treatment, and in turn whether this can lead to meaningful improvements in clinical outcomes for critical care patients.

## Key messages

• In contrast to traditional antibiograms, WISCAs display the likelihood of coverage for a specific infectious syndrome (rather than individual pathogens), and also take into account the potential for poly-microbial infections and the use of multi-drug regimens

• WISCAs can be constructed based on local ecology data for common ICU infections such as VAP or CRBSI

• In this retrospective cohort study, only 1/3 of patients received adequate coverage by 12 h, 1/2 of patients 24 h and only 3/4 of patients by 48 h from culture collection

• Use of WISCA-guided double antibiotic therapy regimens could potentially have resulted in adequate coverage rates +56%, +42% and +18% higher than current care at 12-h, 24-h and 480 h time points

• Prospective research is needed to determine whether actual provision of WISCA antimicrobial prescribing-aids to ICU clinicians are associated with improvements in timely and rational antimicrobial treatment

## Abbreviations

CRBSI: catheter-related bloodstream infection; IDSA: Infectious Diseases Society of America; MODS: multi-organ dysfunction score; SHSC: Sunnybrook Health Sciences Centre; VAP: ventilator-associated pneumonia; WISCA: weighted incidence syndromic combined antibiogram.

## Competing interests

The authors declare that they have no competing interests.

## Authors’ contributions

ND conceived the research question, conducted part of the statistical analysis, and drafted part of the manuscript. VR extracted data from medical records and drafted part of the manuscript. SS extracted data from the medical records, contributed to statistical analysis, and revised the manuscript. ME extracted data from electronic records, assisted in data interpretation, and revised the manuscript. LP assisted in data interpretation, and revised the manuscript. SW assisted in data interpretation and revised the manuscript. All authors read and approved the final manuscript.
